# Genome-wide association studies reveal novel QTLs for agronomic traits in soybean

**DOI:** 10.3389/fpls.2024.1375646

**Published:** 2024-05-14

**Authors:** Dongwei Han, Xi Zhao, Di Zhang, Zhen Wang, Zhijia Zhu, Haoyue Sun, Zhongcheng Qu, Lianxia Wang, Zhangxiong Liu, Xu Zhu, Ming Yuan

**Affiliations:** ^1^ Qiqihar Branch of Heilongjiang Academy of Agricultural Science, Qiqihar, Heilongjiang, China; ^2^ Heilongjiang Chinese Academy of Sciences Qiuying Zhang Soybean Scientist Studio, Qiqihar, Heilongjiang, China; ^3^ Biotechnology Institute, Heilongjiang Academy of Agricultural Science, Harbin, Heilongjiang, China; ^4^ Institute of Crop Sciences, Chinese Academy of Agricultural Sciences, Beijing, China; ^5^ Department of Research and Development, Ruibiotech Co., Ltd, Beijing, China

**Keywords:** soybean, GWAS, pod number per plant, grain number per plant, 100-grain weight

## Abstract

**Introduction:**

Soybean, as a globally significant crop, has garnered substantial attention due to its agricultural importance. The utilization of molecular approaches to enhance grain yield in soybean has gained popularity.

**Methods:**

In this study, we conducted a genome-wide association study (GWAS) using 156 Chinese soybean accessions over a two-year period. We employed the general linear model (GLM) and the mixed linear model (MLM) to analyze three agronomic traits: pod number, grain number, and grain weight.

**Results:**

Our findings revealed significant associations between *qgPNpP-98*, *qgGNpP-89* and *qgHGW-85* QTLs and pod number, grain number, and grain weight, respectively. These QTLs were identified on chromosome 16, a region spanning 413171bp exhibited associations with all three traits.

**Discussion:**

These QTL markers identified in this study hold potential for improving yield and agronomic traits through marker-assisted selection and genomic selection in breeding programs.

## Introduction

Cultivated soybean (*Glycine max L.*) is a principal oilseed crop that is grown globally for its significant contribution to edible protein and oil production (Zong et al., 2017). Despite the success of the revolution in enhancing the yields of rice, wheat, and maize, comparatively lower progress has been made in improving soybean yields (Liu et al., 2020). The global objective of soybean breeders, particularly in China, is to develop genotypes that possess increased yield potential. In soybean, pod number, grain number, and grain weight exist a substantial positive association with grain yield. However, when there is an excessive number of pods and seeds, as well as when the seeds become heavier, soybean plants become more vulnerable to lodging. This can have a detrimental effect on the overall crop yield. Therefore, it is important to pursue a balance between these traits and grain yield in order to optimize the productivity of soybean crops (Zhang et al., 2015; Ning et al., 2018). Additionally, it is worth noting that pod number, grain number, and hundred grain weight are controlled by multiple loci/genes and can be influenced by interactions between genotype and environment. Therefore, it is crucial to consider both genetic factors and environmental conditions when aiming to achieve an optimal balance between these traits and grain yield in soybean crops (Lamichhane et al., 2020; Happ et al., 2021). Both conventional breeding and marker-assisted breeding (MAB) approaches have been utilized to enhance yield traits in soybean crops (Raju et al., 2018). Recent studies have demonstrated that MAB approaches are particularly effective for traits that are sensitive to environmental conditions, such as seed germination, seedling development, and maturity (Saminadane et al., 2024; Xu et al., 2024). By utilizing a specially designed marker primer design program, MAB demonstrates significant potential in molecular breeding, offering a promising avenue for the development of improved crop varieties with desired traits (Xia et al., 2023). Hence, understanding the genetic architecture of yield-related traits is crucial in effectively utilizing marker-assisted breeding (MAB) for the development of high-yielding soybean varieties.

Genome-wide association studies (GWAS) have emerged as a highly effective approach for identifying alleles/QTLs linked to specific traits with high resolution (Zhang et al., 2021). With the advancements in sequencing-based genotyping technologies, GWAS has become increasingly popular in crop genetics research (Zhang et al., 2016, 2022). Numerous studies conducted on various plant species, including rice, maize, wheat, soybean, potato, cucumber, and tomato, have demonstrated the ability of GWAS to uncover associations between marker-trait associations (MTAs) and effectively identify the underlying genes (Zhao et al., 2011; Wang et al., 2016; Yan et al., 2019; Torkamaneh et al., 2020; Gao et al., 2021; Zhou et al., 2021; Liang et al., 2022; Luo et al., 2022; Zhao et al., 2023; Sun et al., 2022a; Sun et al., 2022b; Wang et al., 2015, 2018; Jhon et al., 2023; Zhu et al., 2023). In soybean, multiple research studies have specifically investigated the genetic basis of yield (Dong et al., 2021; Zhao et al., 2021; Bhat et al., 2022). Over the past 20 years, more than 3000 quantitative trait loci (QTLs) have been identified through GWAS in soybean (https://www.soybase.org/). These QTLs are spread across the 20 chromosomes of the soybean genome. However, the lack of effective utilization of the three yield-related QTLs (pod number, total seed number and one-hundred grain weight) has been a persistent obstacle in the development of improved soybean varieties with desirable yield traits.

The utilization of whole genome sequencing data allows for the precise mapping of QTLs associated with agronomic traits. In this study, we conducted a GWAS to identify significant MTAs, candidate genes, and QTLs in soybean cultivars selected from major soybean growing regions in China, which holds great potential for the development of enhanced soybean varieties with targeted yield characteristics through a QTL-based breeding approach.

## Materials and methods

### Experimental materials and cultivate management

A total of 156 soybean genotypes, including 100 accessions selected from germplasm collection by Dr. Lijuan Qiu’s laboratory at the Chinese Academy of Agricultural Sciences and 56 cultivars from Qiqihar Branch of Heilongjiang Academy of Agricultural Science. These genotypes were originally from 3 provinces in China (79, 50.6%) and 10 states in the United States (77, 49.4%), representing a wide range of genetic diversity within and outside China. Experiments were carried out in Qiqihar (123.685996°E, 47.274543°N) during the years 2020 and 2021, utilizing a single-row plot system with 3-meter-long rows spaced at 0.5-meter intervals. The field trials followed a randomized complete block design and were conducted across multiple testing environments, with each test environment containing three replicates. Throughout the growing season, field management practices adhered to standard cropping system protocols, including fertilization, weed management, and insecticide fungicide application.

### Trait identification and statistical analysis

The assessment of all traits from 10 randomly selected plants in each line was conducted after reaching full maturity. All plants and pods derived from conventionally developed plants were gathered for trait examination. Specifically, three traits related to yield were analyzed, including pod number per plant (PNpP), grain number per plant (GNpP), and 100-grain weight (HGW). The PNpP is calculated by counting all pods on 10 randomly selected soybean plants, including both mature and immature pods. The GNpP is the number of seeds contained in all pods of 10 randomly selected soybean materials, divided by 10 to obtain the average number of grains per plant. The statistical method of HGW is the value obtained by randomly selecting 10 soybean materials and then selecting and weighing 100 seeds from the seeds obtained. All these traits were subsequently measured in the laboratory, or determined through electronic weighing. The broad-sense heritability (*H* ^2^) was calculated following a previously reported method (Wyman and Baker, 1991). Statistical analyses of obtained data were calculated by using GraphPad Prism 7.0.

### DNA extraction and SNP Genotyping

DNA samples extracted by CTAB method from 156 accessions were genotyped for SNPs using a soybean 200K array. This array named “ZDX1”, which is a high-throughput SNP genotyping chip developed jointly by the Institute of Crop Sciences of Chinese Academy of Agriculture Science and Beijing Compass Biotechnology Co., Ltd., using the Illumina platform for customization (Sun, et al., 2022c). And a total of 158959 high-quality SNPs were used for association mapping.

### Linkage disequilibrium analysis and population structure

We conducted genome-wide LD analysis using Plink 2 (Chang et al, 2015) with *R* ^2^ < 0.2 as the threshold to identify 3026 unlinked loci. The population structure analysis was conducted using software FastStructure (Raj et al., 2014). Then analysis the kinship applying the software TASSEL version 5.0 (Bradbury et al., 2007).

### Genome-wide association analysis

Two models, mixed linear model (MLM) and general linear model (GLM), were employed to investigate associations between genotypic and phenotypic data in TASSEL version 5.0. Briefly, the MLM approach was used to account for both population structure and kinship matrix, which are jointly incorporated via the Q+K approach for enhanced statistical power (Yu et al., 2006). On the other hand, GLM was utilized to analyze individual location datasets using a least square fixed effect model with Q acting as a covariate with flexible assumptions.

## Results

### Phenotypic analysis of three yield-related traits

**Figure 1:**
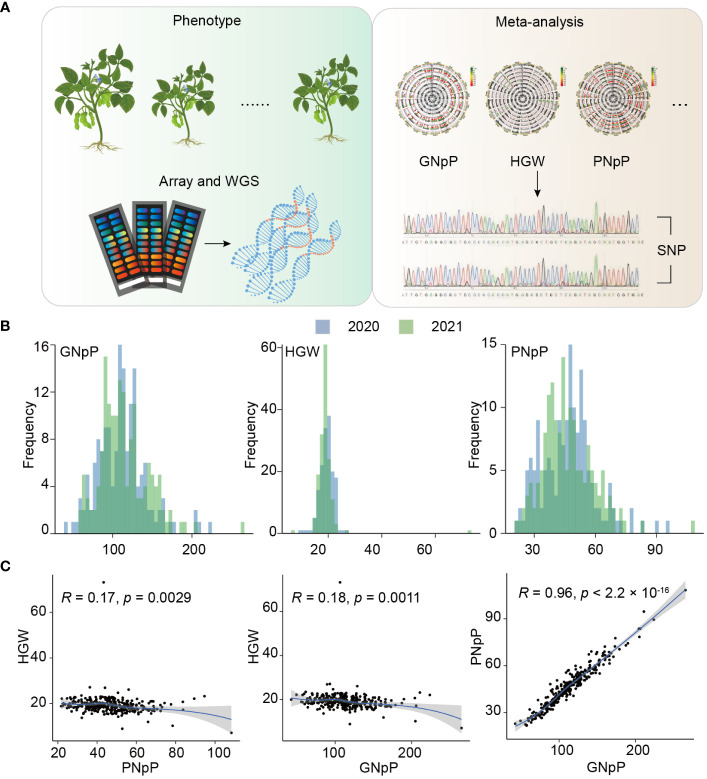
Schematic and statistics of the Experiment. **(A)** Workflow of genotyping by sequencing. **(B)** Frequency distribution of three yield-related traits over two years. **(C)** Phenotypic correlations between three traits GNpP, HGW and PNpP.

To explore loci associated with agronomic traits, a total of 156 soybean landraces were cultivated over two consecutive years, and an investigation of three yield-related traits was conducted (**Figure 1A**). The frequency distribution of the three yield-related traits across the two years showed a continuous distribution in the GWAS panel of 156 soybean landraces, with a wide range of variation (**Figure 1B**). GNpP exhibited 7-fold variation, ranging from 41 to 307, with an average of 112.9 ± 20.1. HGW and PNpP showed approximately 4-fold and 7-fold differences, ranging from 6.8 g to 28.2 g and 18 to 128g, respectively. The frequency distribution of the three yield-related traits displayed an approximately normal distribution except for a few materials that showed large deviation. The broad-sense heritability (*H* ^2^) was determined for each of the three traits. All traits presented an *H* ^2^ above 54%, suggesting that genetic effects play a predominant role in the phenotype variation of these traits (**Supplementary Table 1**). The results of the phenotypic correlation analysis revealed strong correlations between PNpP and GNpP, while other traits exhibited no significant correlations. This suggests that the genetic-associated loci are likely to be similar between PNpP and GNpP, while the other traits are not influenced in a correlated manner (**Figure 1C**).

### SNP profile

**Figure 2:**
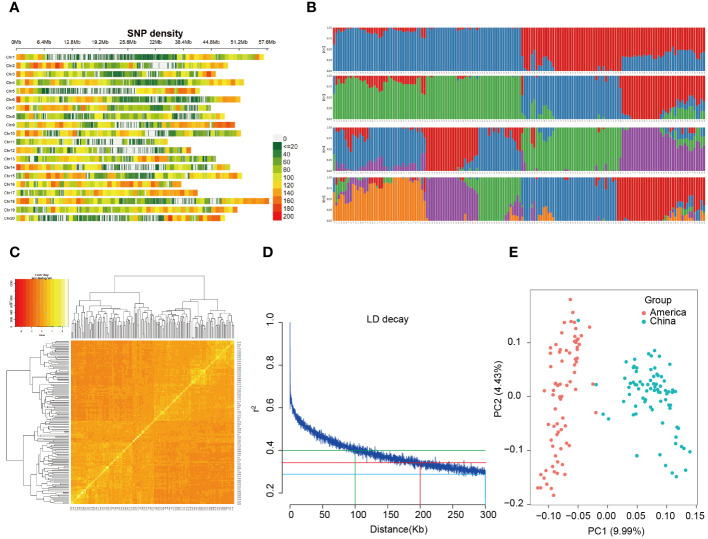
SNP distribution and population structure of 156 soybean landraces. **(A)** Distribution of 80,163 SNP markers across 20 soybean chromosomes for GWAS. A density distribution map of SNPs across the entire genome with bins of 1 Mb. **(B)** Population structure analysis of 156 materials. **(C)** Heatmap of the kinship matrix of the 156 soybean accessions. **(D)** Scatter plot of pairwise SNPs showing genome-wide linkage disequilibrium (LD) decay. The red curve line represents the smoothing spline regression model fitted to LD decay. The vertical red line indicates the genetic distance (200 Kb) at which the LD half-decay (r^2 = ^0.35, the horizontal blue line) intersect with the LD decay curve. **(E)** Principal component analysis (PCA) of the population structure. Distribution of the accessions in the association panel under PC1 and PC2.

A total of 80163 high-quality SNPs (MAF > 0.05, missing rate < 20%) were used for a GWAS of the three traits, with an average marker density of 11.47 kb/SNP at the genome wide scale. The lowest marker density (17.10 kb/SNP) was found on chromosome 1, and the highest marker density (8.55 kb/SNP) was found on chromosome 16. Furthermore, SNPs exhibit a predominant enrichment in sub telomeric regions, particularly in areas distal to the centromeres (**Figure 2A**).

### Population structure of 156 soybean materials

To elucidate the most promising genetic variations and contribute to a comprehensive understanding of the genetic underpinnings of the trait, we conducted a structure analysis. The 156 genotypic materials were stratified into two subpopulations based on the results obtained from the K value (**Figure 2B**). The kinship analysis revealed that the soybean materials utilized in this investigation originated from two primary lineages, further substantiating the existence of dual ancestors for the 156 soybean materials (**Figure 2C**). Examination of the linkage disequilibrium (LD) decay rates with the high-quality SNPs demonstrated that the decay curves of LD exhibited a discernible pattern dependent on distance, revealing steeper decay at longer distances. Furthermore, beyond a marker distance of 5 Mb, the *r* ^2^ value generally remains below 0.1. Precisely, at a distance of 100 kb, the LD exhibited decay with an *r* ^2^ value of 0.4, signifying a notably robust LD correlation among proximate variants. At 200 kb, the LD decayed with an *r* ^2^ value of 0.35, denoting a moderate level of LD correlation between adjacent variants. Lastly, at 300 kb, the LD decayed with an *r* ^2^ value of 0.2, indicating a diminished level of LD correlation between nearby variants. These outcomes correspond to a physical distance of approximately 200 Kb. Consequently, we performed QTL anchoring using a 200 Kb window, specifically focusing on the region extending 100 kb upstream and downstream of the MTA (**Figure 2D**). To discern the degree of SNP variation among the materials, a cluster analysis was executed through principal component analysis (PCA). The results indicated that the 156 materials could be classified into two subgroups based on PC1 (**Figure 2E**), constituting 9.99% of the total variance. This suggests that the 156 soybean materials can be delineated into two subgroups, reflecting a composite of two ancestral populations. The geographical origins of the 156 soybean landraces were the foreign region and domestic region.

### Genome wide association analysis of four yield-related traits

**Figure 3:**
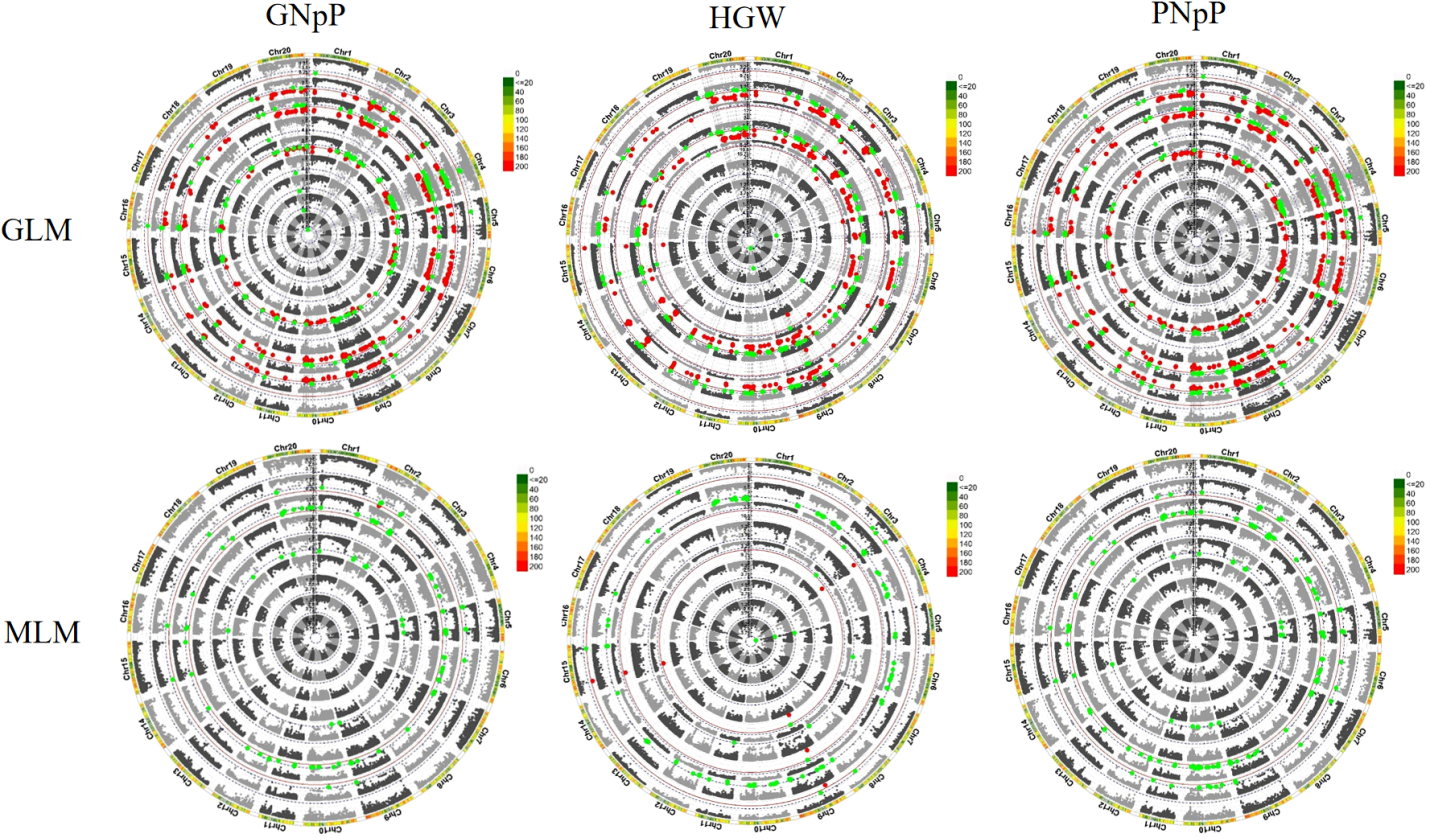
Manhattan plot displays the SNPs significantly associated with agronomic traits detected in GWAS analysis. The data consists of the testing results of population materials in 2020 and 2021, and analysis was conducted using the GLM and MLM modules. The soybean materials are subjected to three repeated experiments every year, and the average of the three repeated experiments each year is taken (denoted as “m”). The circular Manhattan plot represents the values from the center to the outer 1-9 circles as follows: 2020 (m), 2020 (1), 2020 (2), 2020 (3), 2021 (m), 2021 (1), 2021 (2), 2021 (3), and BLUE value of two years. Each colored dot represents a SNP.

To identify significant SNPs associated with target traits, we employed two Genome-Wide Association Study (GWAS) models, namely the General Linear Model (GLM) and the Mixed Linear Model (MLM), and FDR correction, for the analysis of high-quality SNPs within a dataset comprising 156 soybean germplasms. The significant threshold value for the association between SNP and traits were determined by -log10 (P) >4.78, which is equivalent to P <0.5/3026, for GLM, MLM and FDR correction. Quantile–quantile plots are employed to evaluate whether the distribution of observed p-values from statistical tests deviates from the expected distribution. Deviations from the diagonal suggest potential deviations from the null hypothesis, indicating possible associations between genetic variants and the trait (**Supplementary Figure 1**). The Manhattan plots result showed that a few sets of significant SNPs associated with GNpP, HGW and PNpP was detected in 2020 by the two models. The data from 2021 found a few numbers of SNPs with false positives. Based on all the result, SNPs, that were significantly tested at least two times for each trait, were regarded as MTAs. Specifically, in the GLM, a total of 628 QTLs were identified, consisting of 205 QTLs related to GNpP, 206 QTLs related to HGW and 217 QTLs related to PNpP. In contrast, the MLM approach detected a total of 52 QTLs, with 15 QTLs related to GNpP, 9 QTLs related to HGW and 28 QTLs related to PNpP. The method of MLM eliminated lots of false positive SNPs by comparison. Among these QTLs, two or three QTLs can form a colocalization region, and thirteen colocalization regions in all were found. These colocalization regions distributed on 9 of 20 soybean chromosomes, and most of them associated with both PNpP and GNpP. Only the region on chromosome 6 associated with PNpP, GNpP and HGW (**Figure 3**).

### Prediction of candidate genes

**Figure 4:**
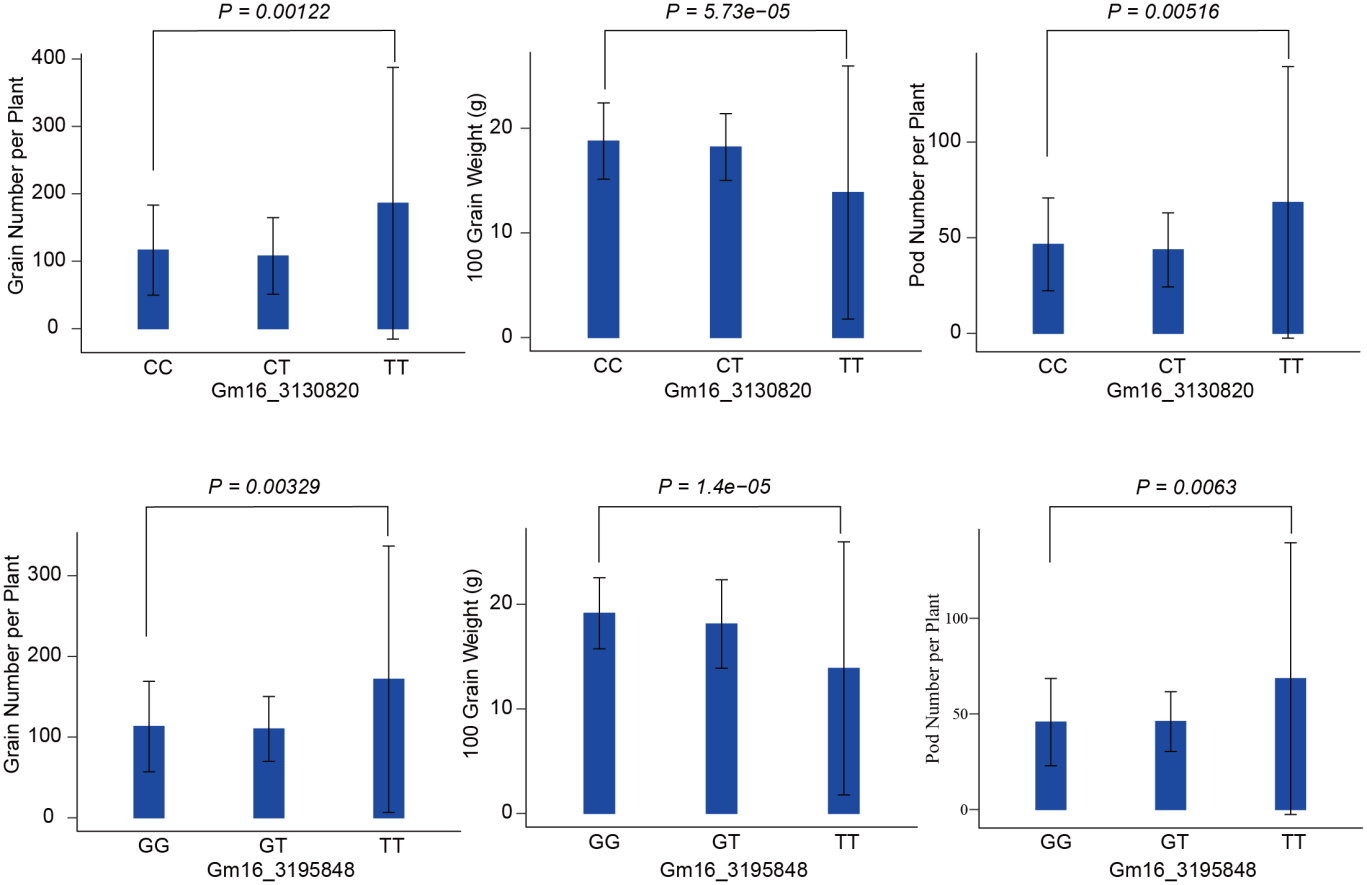
Phenotypic differences between accessions carrying different alleles. They are the allele effects for the marker Gm16_3130820 and Gm16_3195848 of GNpP, HGW and PNpP in soybean. GNpP means grain number per plant, HGW means 100 grain weight, and PNpP means pod number perplant.

There are three QTLs named *qgGNpP-89*, *qgHGW-85*, and *qgPNpP-98*, associated with GNpP, HGW and PNpP individually, which are in a specific region spanning from 3030820 to 3424009 base pairs on chromosome 16 was identified by GLM. This region contained ten marker sites carrying different alleles, and showed a significant positive correlation between them. More notably, seven of these marker loci were related to GNpP, HGW and PNpP at the same time (**Table 1**). We were particularly interested in the markers with large effects, such as marker Gm16_3130820 and Gm16_3195848 on chromosome 16. Compared with the alternative alleles, the GNpP and PNpP of the materials carrying the favorable allele (TT) at Gm16_3130820 was higher than the materials carrying the unfavorable allele (CC), the HGW of the materials carrying the favorable allele (CC) at Gm16_3130820 was higher than the materials carrying the unfavorable allele (TT). Otherwise, the GNpP and PNpP of the materials carrying the favorable allele (TT) at Gm16_3195848 was higher than the materials carrying the unfavorable allele (GG), the HGW of the materials carrying the favorable allele (GG) at Gm16_3195848 was higher than the materials carrying the unfavorable allele (TT) (**Figure 4**). It’s also worth mentioning that three of these seven markers, GM16_3130820, GM16_3195848, and GM16_3260654, were found to be located on three candidate genes, *Glyma.16G033100*, *Glyma.16G034100*, and *Glyma.16G034600*, respectively. Candidate genes *Glyma.16G033100* is responsible for encoding the S-adenosylmethionine-binding subunit, which plays a crucial role in various biochemical processes. It associated with dividing tissues, particularly reproductive organs (Zhong et al., 2008) such as floral organ number and size (Bodi et al., 2012), tiller bud formation (Yu et al., 2021), and shoot meristems. It also influenced seed development with protein and the starch synthesis-related pathway enriched in the later stages (Li et al., 2022). *Glyma.16G034100* codes for a zinc finger and C3HC4 type (ring finger), a protein domain known for its ability to bind zinc ions and regulate gene expression. In rice, research showed that *FRRP1* probably regulates flowering time and yield potential by affecting histone H2B monoubiquitylation (Du et al., 2016). The *AtYY1* gene is a negative regulator of the *Arabidopsis* ABA response network in *Arabidopsis* (Li et al., 2016). And these proteins also can regulate seed weight (Barmukh et al., 2021), tiller number (Zhou et al., 2017), and seed size and plant height in crop plants (Du et al., 2016). Lastly, *Glyma.16G034600* is associated with the protein tyrosine kinase, a protein plays a fundamental role in regulation of most cellular activities. They have been shown to impact the seed and yield of crops (*Arabidopsis*, rice, sunflower, potato and so on) through various pathways, such as regulation of carbon supply (Zheng et al., 2010), development of embryo (Thakur and Bhatla, 2014; Mazin et al., 2019), and cell proliferation (Zhang et al., 2020), BR signaling and control (Tian et al., 2021).

**Table 1:** Traits co-location and associated markers.

Traits	QTLs	chromosome	RegionStart	RegionEnd	LOD	R^2^	Markers
GNpP	*qgGNpP-89*	16	3010838	3424009	4.81 - 6.69	12.19 - 16.86	Gm16_3110838; Gm16_3130820; Gm16_3169888; Gm16_3195848; Gm16_3203580; Gm16_3260654; Gm16_3271292; Gm16_3283717; Gm16_3304635; Gm16_3324009
HGW	*qgHGW-85*	16	3030820	3424009	4.99 - 5.90	11.78 - 13.32	**Gm16_3130820**; Gm16_3169888; Gm16_3195848; **Gm16_3203580**; **Gm16_3260654**; **Gm16_3271292**; **Gm16_3283717**; **Gm16_3304635**; **Gm16_3324009**
PNpP	*qgPNpP-98*	16	3030820	3424009	4.91 - 6.82	12.05 - 17.06	Gm16_3130820; Gm16_3203580; Gm16_3260654; Gm16_3271292; Gm16_3283717; Gm16_3304635; Gm16_3324009

The seven marker loci in bold: Gm16_3130820, Gm16_3203580, Gm16_3260654, Gm16_3271292, Gm16_3283717, Gm16_3304635, and Gm16_3324009, were related to GNpP, HGW and PNpP at the same time.

In the region of co-localization, a total of ten candidate genes were found, encoding five kinases (protein kinase, glycosyl hydrolase, m^6^A methyl-transferase, protein tyrosine kinase and ubiquitin-conjugating enzyme), two domains (cyclin-like domain and zinc finger and ring finger) and one repeat (PPR repeat) **Table 2**. Most of the candidate genes associated with kinases have relatively long sequences, ranging from approximately 3000 to 10000 base pairs. The candidate gene sequences related to PPR repeat are shorter, falling within the range of 2000 to 3000 bps. On the other hand, the candidate gene sequences associated with the cyclin-like domain are relatively longer, approaching 14000 bps. Lastly, the candidate gene sequences related to the zinc finger protein have lengths less than 1000 bps. Otherwise, some of the candidate genes were closely related to each other, such as candidate genes *Glyma.16G033000* and *Glyma.16G033100* are 5658 bps apart, encoding glycosyl hydrolase family and S-adenosylmethionine-binding subunit, respectively (**Supplementary Tables 3-8**).

**Table 2:** Candidate gene information.

Functional annotation	Gene ID	RegionStart	RegionEnd
protein kinase	*Glyma.16g032700*	3094789	3099121
glycosyl hydrolase family 28	*Glyma.16g033000*	3116063	3122058
S-adenosylmethionine-binding subunit	*Glyma.16g033100*	3127716	3133331
protein tyrosine kinase	*Glyma.16g034600*	3254425	3263715
ubiquitin-conjugating enzyme	*Glyma.16g035000*	3311992	3314979
cyclin-like domain	*Glyma.16g035400*	3348514	3362406
PPR repeat	*Glyma.16g031800*	3017265	3019791
zinc finger and C3HC4 type (ring finger)	*Glyma.16g034100*	3195056	3196022

## Discussion

The quest for increasing soybean yield is a paramount goal for breeders, and the use of GWAS has proven to be an effective method to uncover the genetic components related to soybean yield. Several studies have successfully identified various SNP loci, QTLs, and candidate genes (Hao et al., 2012; Liu et al., 2017; Jing et al., 2018; Hu et al., 2020; Mohsen et al., 2021; Chanditha et al., 2022) associated with soybean yield and its components. In the current study, the focus was on three essential yield-related traits: PNpP, GNpP, and HGW. Using GWAS, a substantial number of SNPs associated with these traits were identified within the soybean population. Most significantly, a co-localization interval on chromosome 16 was discovered, associating with all three traits. This interval contained eight candidate genes involved in crop growth and development, which had not been reported previously.

These candidate genes have diverse functions, such as transcriptional initiation of protein-coding genes (e.g., *Glyma.16g035400*), regulation of genes involved in DNA repair, cell cycle, and apoptosis (e.g., *Glyma.16g031800*), and modulation of alternative RNA splicing (e.g., *Glyma.16g033100*). Furthermore, two candidate genes (*Glyma.16g032700* and *Glyma.16g034600*) were associated with protein kinases, while another (*Glyma.16g033000*) was linked to cell wall metabolism. The ubiquitin-proteasome system (UPS) was also represented in the candidate genes, with *Glyma.16g035000* acting as a ubiquitin-conjugating enzyme (E2), and *Glyma.16g034100* likely functioning as E3 ubiquitin ligases. In summary, the study employed GWAS on both Chinese and foreign soybean varieties, revealing a co-localization locus associated with PNpP, GNpP, and HGW. Within this region, ten candidate genes and seven common markers were identified, with three of the markers mapping to three candidate genes. The proximity of these candidate genes related to yield traits underscores the importance of this locus. Further investigations into the expression patterns of these candidate genes at specific growth stages will help in improving soybean varieties and overcoming yield limitations.

## Data availability statement

The original contributions presented in the study are publicly available. This data can be found here: FigShare, https://figshare.com/articles/dataset/_b_Genome-wide_association_studies_b_b_reveal_novel_b_b_QTLs_b_b_for_b_b_agronomic_traits_b_b_in_soybean_b_/25690689.

## Author contributions

DH: Writing – original draft, Writing – review & editing, Visualization, Validation, Supervision, Software, Resources, Project administration, Methodology, Investigation, Funding acquisition, Formal analysis, Data curation, Conceptualization. XZ: Visualization, Supervision, Resources, Project administration, Methodology, Conceptualization, Writing – review & editing, Writing – original draft, Validation, Software, Investigation, Formal analysis, Data curation. DZ: Writing – review & editing, Project administration. ZW: Writing – review & editing, Project administration. ZZ: Writing – review & editing, Project administration. HS: Writing – review & editing, Project administration. ZQ: Writing – review & editing, Project administration. LW: Writing – review & editing, Project administration. ZL: Writing – review & editing, Conceptualization. XZ: Writing – review & editing, Software, Formal analysis, Conceptualization. MY: Writing – review & editing, Conceptualization.
