# Supplementary material for: Genome-wide association studies reveal novel QTLs for agronomic traits in soybean
Source: Front Plant Sci. 2024 May 14;15:1375646. doi: 10.3389/fpls.2024.1375646 (PMC11132100; doi:10.3389/fpls.2024.1375646)
Supplement: Supplementary file 2 [file DataSheet_1.doc]

**Supporting information to “Genome-wide association studies reveal novel QTLs and candidate genes for yield in soybean”**

Dongwei Han^1,3#^, Xi Zhao^1#^, Di Zhang^1^, Zhen Wang^1^, Zhijia zhu^1^, Haoyue Sun^1^, Zhongcheng Qu^1^, Lianxia Wang^1^, Zhangxiong Liu^1*^, Xu Zhu^2*^, Ming Yuan^1*^

^1^Qiqihar Branch of Heilongjiang Academy of Agricultural Science, Qiqihar, Heilongjiang, China

^2^Shenzhen Branch, Guangdong Laboratory of Lingnan Modern Agriculture, Genome Analysis Laboratory of the Ministry of Agriculture and Rural Affairs, Agricultural Genomics Institute at Shenzhen, Chinese Academy of Agricultural Sciences, Shenzhen, Guangdong, China.

^3^Heilongjiang Chinese Academy of Sciences Qiuying Zhang soybean scientist studio

^#^These authors contributed equally to this work: Dongwei Han, Xi Zhao.

^*^Correspondence: [liuzhangxiong@caas.cn](mailto:liuzhangxiong@caas.cn) (Zhangxiong Liu); zhu.xu@ruibiotech.com (Xu Zhu); [y.m@haas.cn](mailto:y.m@haas.cn) (Ming Yuan)

**Supplemental Figures**


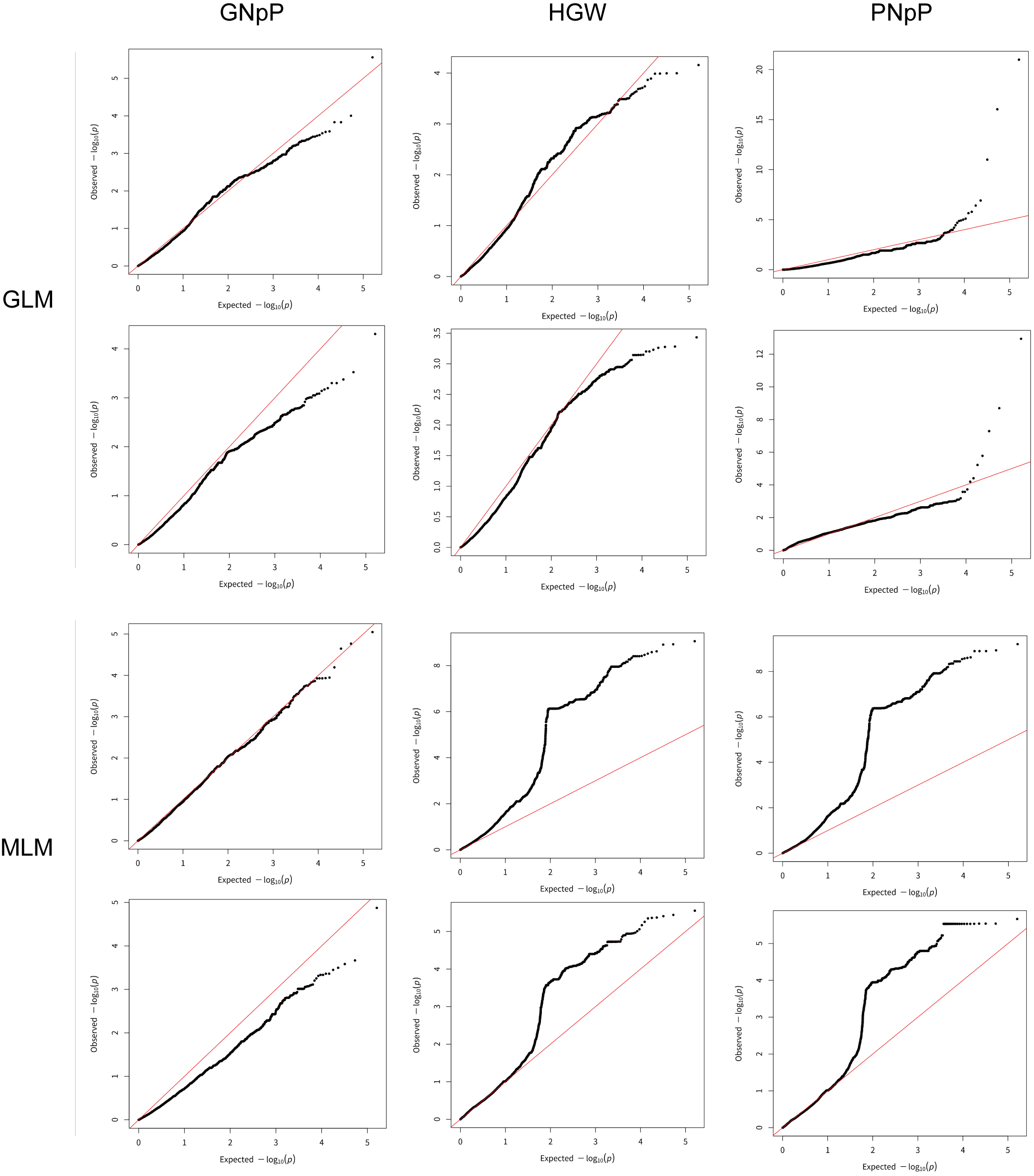
Figure S1.

Q-Q plots showing the trend of change detected in the year 2020 and 2021 GWAS analysis for the agronomic traits. (1) The first and third rows are the results of 2020; (2) The second and fourth rows are the results of 2021. Red line represents expected P values with no association. To evaluate the effectiveness of different models in controlling false associations, correlation analysis was conducted on three traits related to yield. For GNpP, both the GLM and MLM models showed smaller deviations between the observed p-values and the expected p-values assuming no association in 2020 and 2021. This indicates that both models are suitable for analyzing GNpP data samples. For HGW and PNpP, the GLM model showed smaller deviations between the observed p-values and the expected p-values assuming no association, while the MLM model had larger deviations. This suggests that the GLM model is more suitable for analyzing HGW and PNpP data samples. Therefore, the selection of SNP loci will primarily rely on the results obtained from the GLM model.
